# Predictors of the patient-centered outcomes of surgical carpal tunnel release – a prospective cohort study

**DOI:** 10.1186/s12891-016-1046-3

**Published:** 2016-04-27

**Authors:** Catharina Conzen, Michael Conzen, Nicole Rübsamen, Rafael Mikolajczyk

**Affiliations:** Neurosurgical outpatients’ clinic Dr. med. Dr. PH Michael A. Conzen and partners, Bielefeld, Germany; ESME – Epidemiological and Statistical Methods Research Group, Helmholtz Centre for Infection Research, Inhoffenstr. 7, 38124 Braunschweig, Germany; Hannover Medical School, Hannover, Germany

**Keywords:** Carpal tunnel syndrome, Patient-centered outcomes, Nerve conduction velocity

## Abstract

**Background:**

Carpal tunnel syndrome (CTS) causes a substantial burden of disease in society. While CTS can be resolved by surgical carpal tunnel release, it still remains unclear as to what degree outcomes depend on patients’ characteristics. This study assesses patient-centered outcomes after surgical carpal tunnel release in a large outpatient clinic in Germany.

**Methods:**

Patients with CTS were recruited prospectively between August 1^st^ and December 31^st^, 2013. We assessed socio-demographic and psychological factors as well as nerve conduction velocities at baseline (before the surgery) and at three and six months after surgery. We analyzed the improvement of patient-centered outcomes (symptoms and function of the affected hand as well as measures of well-being and subjective quality of life) at the two follow-up time points and investigated if socio-demographic characteristics and CTS-related variables predict the success of the surgery with respect to nerve conduction velocities and patient-centered outcomes by means of analysis of covariance (ANCOVA). Factors influencing the duration of sick leave were investigated by means of Cox regression.

**Results:**

The study sample consisted of 71 CTS cases. Surgical carpal tunnel release generally improved nerve conduction velocity and patient-centered outcomes. Regarding the former, the improvement was proportional to the severity score at baseline. The presence of muscular atrophy in the thenar area at baseline displayed medium size effects for the patient-centered outcomes. Other socio-demographic characteristics and CTS-related variables did not have a strong predictive effect on the improvement of nerve conduction velocity and patient-centered outcomes.

**Conclusions:**

There is a significant improvement of clinical and subjective outcomes after CTS surgery in the outpatient sector. The improvement is largely independent of socio-demographic and clinical characteristics of the patients.

## Background

Carpal tunnel syndrome (CTS) is the most common entrapment neuropathy and, given its socio-economic consequences, it causes a substantial burden of disease in society [1]. It is usually treated by surgical decompression with generally favorable outcomes [2]. In Germany, about 300,000 operations are performed every year, 90 % of them on outpatient basis [3, 4]. The return-to-work interval following carpal tunnel release is extremely variable, ranging from a few days on sick leave to several months. In some studies, 10 to 20 % of the patients did not return to work at all after the surgery [5, 6]. Considering the high incidence of CTS [7], an understanding of factors predicting a poor outcome following open carpal tunnel release is a core objective of recent research. The clinical outcome and duration of sick leave seem to depend on multiple factors. Being female and workplace factors like exposure to force and repetitive tasks as well as lower income and lower support by co-workers predicted a delayed return to work in past studies [6, 8]. Similarly, older age, lower pre-operative motor function, and very severe nerve conduction impairment have been related to a poorer functional outcome after the surgery [9–11]. However, the findings are controversial: Other studies did not reveal any association between age, sex, pre-operative function, and outcome, while indicating that women reported stronger pre-operative symptoms than men [10, 12, 13]. In contrast, older patients have reported fewer subjective complaints than younger patients despite a higher severity of CTS [14]; at the same time, they were less satisfied after surgery [10]. Socioeconomic factors like type of insurance and worker’s compensation status have also been related to a poorer outcome [5, 6, 15].

Every health care system has its own characteristics that might influence outcomes after CTS surgery. Only few studies investigated possible predictors of outcome post-CTS surgery in Germany [4, 16]. The purpose of this prospective study was therefore to assess physiological and patient-centered outcomes after surgical carpal tunnel release in a large outpatient clinic in Germany.

## Methods

### Recruitment

Between August 1^st^ and December 31^st^, 2013, participants were recruited at the neurosurgical outpatients’ clinic Dr. med. Dr. PH Michael A. Conzen and partners, Bielefeld. Patients were eligible if they had pathological nerve conduction difficulties in the median nerve and sufficient communication skills in the German language to fill in the questionnaires. Patients with neurological signs of widespread peripheral neuropathy, attendant arthrosis, tendon pathologies, or inflammatory diseases were excluded from the study. Patients underwent an open carpal tunnel release without tourniquet under local anesthesia by two neurosurgeons according to the standard procedures of the outpatient clinic. Because of the scarcity of such data from Germany, the study was primarily explorative. The targeted sample size was 100 patients to allow for estimation of prevalence at a precision of 10 %. Due to difficulties in recruitment, this number was not reached. However, we think that our study is of sufficient size to describe the main tendencies.

### Measurements

Patients answered questions about socio-demographic and psychological factors as well as medical history, well-being, quality of life, and physical stress due to work (via a modified version of the Latko scale [17]). Quality of life had been measured using the FLZ^M^ questionnaire [18], which expresses life satisfaction as a score ranging from −96 to +120. Patients assessed the severity of their symptoms at baseline (before surgery), at the three-month, and at the six-month follow-up appointment using the Boston Questionnaire (BQ) (slightly modified version, translated to German) [19]. At each visit, they also assessed the amount of their pain on a numeric rating scale of pain (NRS-P, 1 = “no pain at all”, 10 = “worst pain one could imagine” [20]) and their perceived strength in the affected hand on a numeric rating scale of perceived strength (NRS-PS, 1 = “no strength at all, paralyzed”, 10 = “full strength”). Every patient underwent a standardized clinical examination and nerve conduction measurement at all three visits. The clinical examination included a visual inspection for presence of muscular atrophy in the thenar area, functional muscle tests of the median-innervated hand muscles, and tests of sensibility using the WEST neurofilament test [21]. During the examination, the temperature of the hand was kept above 30 ° C. Median and ulnar nerves were examined bilaterally. The standard nerve conduction measurement used at the clinic included measurements of distal motor latencies (DML) to the M. abductor pollicis brevis and orthodromic or antidromic distal sensory latencies (DSL) (wrist – F2/F5) (ulnar and median). Nerve conduction velocity (NCV) results were graded according to the severity scale proposed by Bland [22] (Table 1). Severity was then re-categorized into three categories to divide the patients into equally sized groups: “normal/mild” (severity less than two), “moderate” (severity equals two), and “severe” (severity greater than two).

**Table 1:** Severity scale according to Bland [22] that was used to grade nerve conduction measurement results

0	Normal	DML less than 4.1 ms; normal DSL (DSL less than 2.8 ms)
1	Mild	DML < 4.1 ms; slow DSL (2.8 ≤ DSL < 4.1)
2	Moderate	4.1 ≤ DML < 6.5; DSL any value except for conduction block
3	Severe	4.1 ≤ DML < 6.5 ms; DSL conduction block
4	Very severe	6.5 ≤ DML; DSL any value
5	Extremely severe	DML conduction block; DSL conduction block

*DML* distal motor latencies, *DSL* distal sensory latencies

### Statistical analysis

Socio-demographic characteristics and CTS-related variables were described as frequencies and mean or median values for the total sample as well as the severity levels of CTS. Analyses were focused on NCV and five patient-centered outcomes (BQ score of symptoms, BQ score of function, NRS-PS, WHO-Five well-being index, and subjective quality of life). The improvements of NCV were described as the percentage of patients who had pathological values at baseline and maintained similar pathological values six months after surgery. The association between NCV measured before surgery and six months later was further investigated using a spline model as implemented in R library “gam” (excluding those who had a complete block at any time point). The timeline of improvement for the NCV and the patient-centered outcomes between baseline, three, and six months after surgery was investigated by testing if there was a significant difference between the mean values at the different visits. Linear regression analysis was used to investigate if socio-demographic characteristics, the WHO-Five well-being index (as a measure of depressive symptoms), and CTS-related variables predict the success of the surgery with respect to NCV and patient-centered outcomes. A linear regression model was built for every combination of predictors and outcomes (value at six months after surgery). The change between the two time points is related to the baseline value, a phenomenon commonly known as regression to the mean. Therefore, we adjusted for the baseline value of the outcome in each model (analysis of covariance (ANCOVA) [23]). In addition to the regression coefficients and the 95 % confidence intervals (CI), the effect size was calculated as a measure of how much variation in the given outcome at six months could be explained by the given predictor. The calculation of effect sizes was based on partial eta squared [24]. A partial eta squared equal to or greater than 0.01 presents a small effect, equal to or greater than 0.06 presents a medium effect, and equal to or greater than 0.14 presents a strong effect [24]. Finally, a Cox regression model was used to assess characteristics associated with the duration of sick leave.

## Results

### Description of the study population

There were 71 cases of CTS surgery in the study sample (three patients had had surgery on both hands) (Table 2). More women had had surgery compared to men (63.4 % vs. 36.6 %, respectively; *p* = 0.03).

**Table 2:** Baseline characteristics of the patients by severity of CTS

	Total: n (%)*	Normal/mild: n (%)*	Moderate: n (%)*	Severe: n (%)*	*P* value^a^
	*N* = 71	*N* = 15	*N* = 28	*N* = 25	
Age					
Median (interquartile range)	50.5 years (40–60)	49 years (31–53)	48 years (38.5 –57)	56.5 years (49.5 –67.5)	0.01
Sex					<0.001
Female	45 (63.4)	12 (80)	24 (85.7)	8 (32)	
Male	26 (36.6)	3 (20)	4 (14.3)	17 (68)	
Smoking status					0.6
Non-smoker	35 (50)	7 (46.7)	12 (44.4)	14 (56)	
Current smoker	21 (30)	5 (33.3)	11 (40.7)	5 (20)	
Former smoker	14 (20)	3 (20)	4 (14.8)	6 (24)	
Education					0.44
Low^b^	55 (83.3)	10 (76.9)	24 (92.3)	18 (75)	
Intermediate^c^	9 (13.7)	2 (15.4)	2 (7.7)	5 (20.8)	
High^d^	2 (3.0)	1 (7.7)	0 (0)	1 (4.2)	
Job type (Latko scale)					0.33
Non-repetitive (1–7 points on Latko scale)	24 (53.3)	8 (72.7)	9 (52.9)	7 (43.8)	
Repetitive (8–10 points on Latko scale)	21 (46.7)	3 (27.3)	8 (47.1)	9 (56.3)	
Body mass index					
Median (interquartile range)	28.3 (24.3–32.0)	23.9 (22.0–31.7)	27.4 (23.5–29.0)	29.4 (28.4–35.6)	0.006
WHO-Five well-being index					
Median (interquartile range)	56 (32–64)	42 (32–60)	56 (32–64)	56 (40–76)	0.28
Handedness					0.53
Right-handed	65 (94.2)	15 (100)	24 (92.3)	23 (92)	
Left- or mixed-handed	4 (5.8)	0 (0)	2 (7.7)	2 (8)	

*Differences to total N due to missing values

^a^Chi-squared test for categorical variables and Kruskal-Wallis test for continuous variables

^b^Low level of vocational or secondary education (less than 12 years of school education and/or completed apprenticeship)

^c^Intermediate level of vocational or secondary education (at least 12 years of school education and/or degree of a specialized vocational school)

^d^University training (Bachelor’s and higher academic level)

The distribution of sex and the median BMI differed among the three categories of severity (Table 2). There were no differences among these categories regarding either other socio-demographic variables (Table 2) or clinical characteristics associated with CTS at baseline (Table 3).

**Table 3:** Carpal tunnel syndrome related variables by severity of CTS

	Total: n (%)*	Normal/mild: n (%)*	Moderate: n (%)*	Severe: n (%)*	*P* value^a^
	*N* = 71	*N* = 15	*N* = 28	*N* = 25	
Affected hand					0.22
Left hand	8 (11.3)	1 (6.7)	5 (17.9)	2 (8)	
Right hand	14 (19.7)	6 (40)	4 (14.3)	4 (16)	
Both hands	49 (69)	8 (53.3)	19 (67.9)	19 (76)	
Dominant hand affected					0.42
Yes	63 (88.7)	14 (93.3)	23 (82.1)	23 (92)	
No	8 (11.3)	1 (6.7)	5 (17.9)	2 (8)	
Family history of CTS					0.13
No relative is affected	40 (58)	7 (46.7)	14 (51.9)	18 (75)	
One or more relatives are affected	29 (42)	8 (53.3)	13 (48.1)	6 (25)	
Thumb opposition					0.18
Yes	33 (51.6)	10 (66.7)	14 (56)	9 (37.5)	
No	31 (48.4)	5 (33.3)	11 (44)	15 (62.5)	
Presence of muscular atrophy in thenar area					0.48
Yes	25 (35.7)	4 (26.7)	9 (36)	11 (45.8)	
No	40 (57.1)	11 (73.3)	16 (64)	13 (54.2)	
Sick leave^b^					
Median (interquartile range)	35 days (28–42)	35 days (29.5–47)	35 days (28–42)	31.5 days (21–35)	0.29

*Differences to total N due to missing values

^a^Chi-squared test for categorical variables and Kruskal-Wallis test for continuous variables

^b^25 of 71 cases had no data on sick leave because the patients were currently unemployed

### Improvement of NCV

**Fig. 1:**
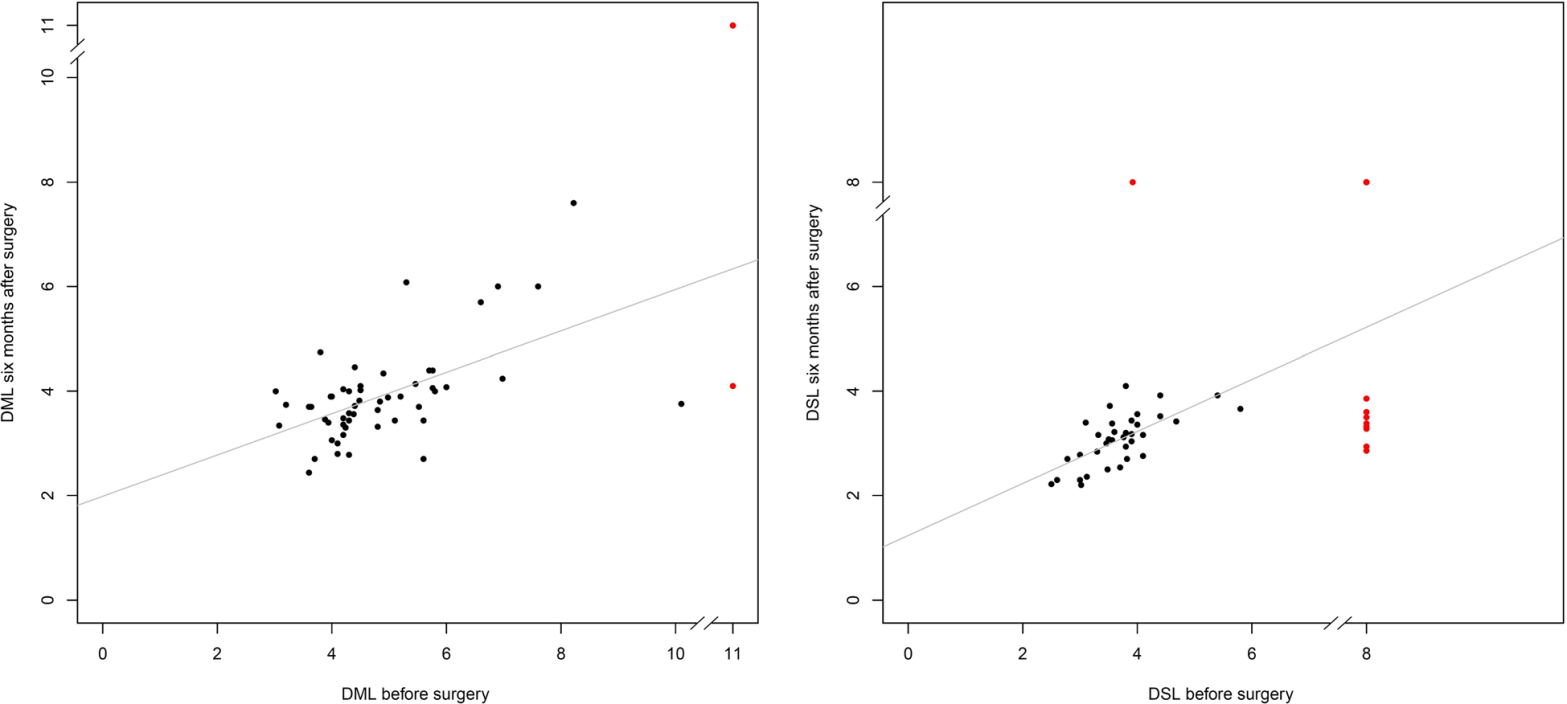
Association between the pre-surgical measurement and measurement taken six months later. DML: distal motor latencies. DSL: distal sensory latencies. Red: Conduction block (replaced by rounded maximum DML or rounded maximum DSL, respectively; excluded from linear regression analysis)

At baseline, 78.0 % of the patients had moderate to extremely severe scores of severity (based on NCV). Six months after surgery, this number had dropped to 32.2 %. The mean NCV had improved significantly post-surgery, and the association between the pre-surgical value and the value six months after surgery indicated a proportional improvement with relation to the baseline measurement (Fig. 1 – while a more complex model was allowed by the statistical procedure, the linear model was supported by the data).

### Overall effects of surgical release of the carpal tunnel syndrome

**Fig. 2:**
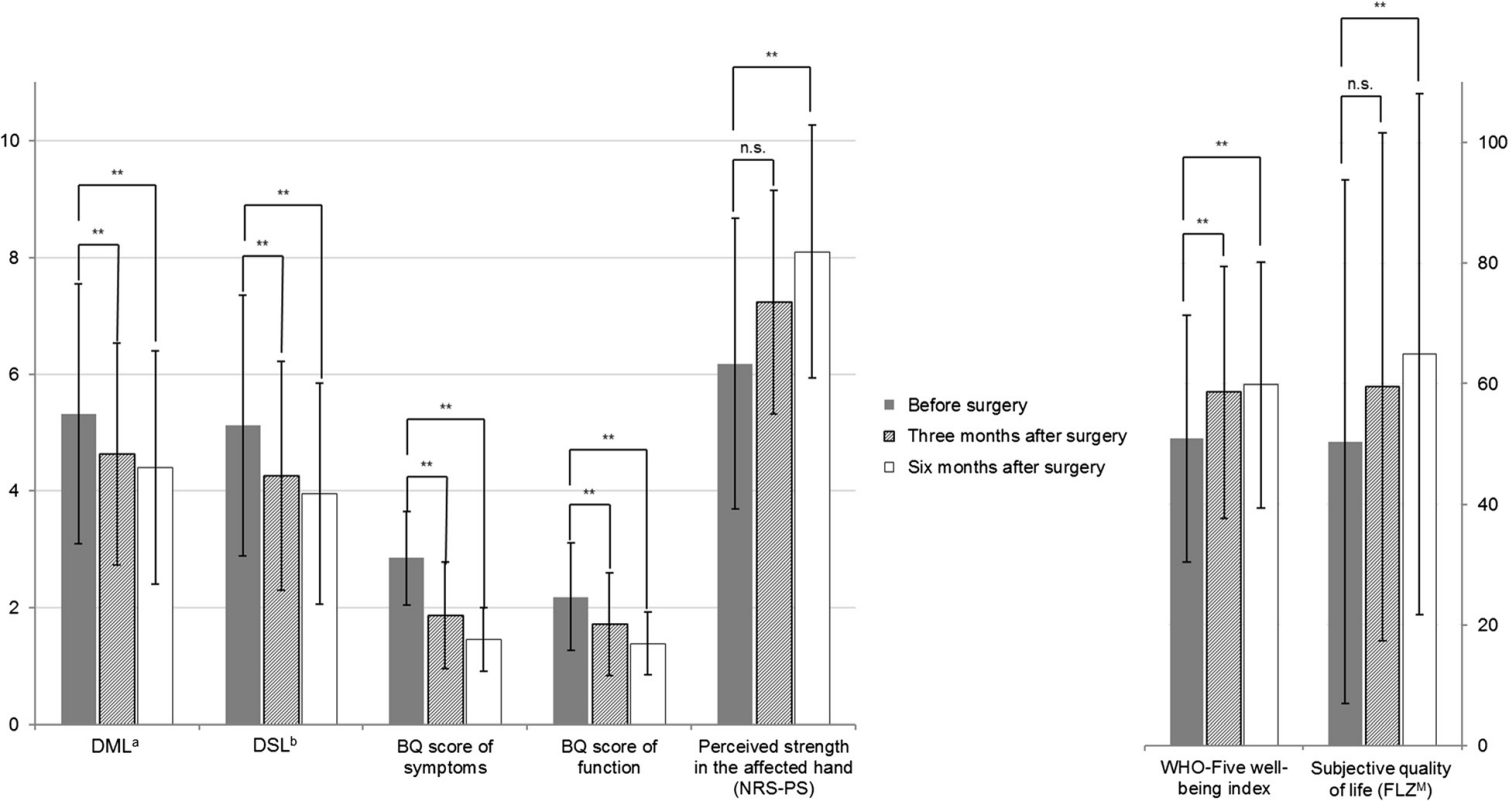
Nerve conduction velocity and patient-centered outcomes at baseline (before surgery) and at three and six months after surgery. BQ: Boston questionnaire (5 points is the worst and 1 point the best score for each scale). DML: distal motor latencies. DSL: distal sensory latencies. ^a^ Conduction block replaced by rounded maximum DML (in *n* = 6; 4; 4 values for time point 1; 2; 3, respectively). ^b^ Conduction block replaced by rounded maximum DSL (in *n* = 22; 13; 10 values for time point 1; 2; 3, respectively). ** Significant (t-test, *p* < 0.01). n.s. Not significant

Surgery generally improved the studied outcomes. Improvements in NCV, BQ scores, and WHO-Five well-being index occurred mainly in the first three months, with little progress observed thereafter. Improvements in perceived strength in the affected hand and subjective quality of life continued beyond three months (Fig. 2).

### Predictors of improvement

Most variables did not have a strong predictive effect on improvements of CTS in the univariate analyses (Table 4). The numbers in Table 4 are interpreted as follows: The betas (linear regression coefficients) represent the expected change in the outcome for a one-unit change in the predictor. In the case of categorical predictors, the betas represent the expected change in the outcome for switching from the reference category to the reported category. The partial η^2^ represents the percentage of the variation in the outcome explained by the predictor.

**Table 4:** Effect sizes of socio-demographic and clinical variables with respect to patient-centered outcomes of surgical release of carpal tunnel syndrome

	Distal motor latencies			Distal sensory latencies			BQ score of symptoms			BQ score of function			NRS-PS			WHO-Five well-being index			Subjective quality of life		
	Beta (95%CI)	P	Partial η^2^	Beta (95 % CI)	P	Partial η^2^	Beta (95 % CI)	P	Partial η^2^	Beta (95 % CI)	P	Partial η^2^	Beta (95 % CI)	P	Partial η^2^	Beta (95 % CI)	P	Partial η^2^	Beta (95 % CI)	P	Partial η^2^
Age	0.09 (−0.15, 0.34)	0.45	0.01	0.32 (0.01, 0.64)	0.05	0.08	0.1 (0, 0.2)	0.05	0.07	0.05 (−0.06, 0.17)	0.34	0.02	−0.43 (−0.83, −0.03)	0.03	0.10	−0.12 (−3.56, 3.32)	0.95	0.00	−3.59 (−11.09, 3.9)	0.34	0.02
Sex		0.05	0.07		0.05	0.07		0.29	0.02		0.26	0.03		0.04	0.09		0.10	0.05		0.91	0.00
Female	Ref			Ref			Ref			Ref			Ref			Ref			Ref		
Male	0.82 (0.01, 1.63)			0.98 (0, 1.96)			0.16 (−0.14, 0.47)			0.19 (−0.15, 0.53)			−1.27 (−2.47, −0.06)			8.48 (−1.65, 18.62)			1.21 (−19.41, 21.83)		
Smoking status		0.08	0.09		0.61	0.02		0.06	0.10		0.34	0.04		0.18	0.07		0.67	0.02		0.92	0.003
Non-smoker	Ref			Ref			Ref			Ref			Ref			Ref			Ref		
Current smoker	−0.22 (−1.07, 0.63)			0.11 (−0.91, 1.13)			−0.37 (−0.71, −0.04)			−0.27 (−0.66, 0.13)			1.15 (−0.24, 2.53)			4.57 (−6.61, 15.76)			1.19 (−21.92, 24.29)		
Former smoker	0.95 (−0.06, 1.95)			0.57 (−0.63, 1.77)			−0.29 (−0.69, 0.12)			−0.15 (−0.58, 0.28)			0.89 (−0.64, 2.43)			3.42 (−9.69, 16.53)			−4.56 (−32.75, 23.64)		
Education		0.19	0.06		0.38	0.04		0.82	0.01		0.43	0.04		0.85	0.01		0.31	0.05		0.79	0.01
Low	Ref			Ref			Ref			Ref			Ref			Ref			Ref		
Intermediate	−0.78 (−1.86, 0.3)			−0.64 (−1.88, 0.6)			0.06 (−0.38, 0.49)			−0.28 (−0.78, 0.23)			−0.44 (−2.1, 1.23)			10.6 (−3.99, 25.2)			1.14 (−28.85, 31.13)		
High	0.9 (−1.16, 2.96)			0.89 (−1.42, 3.19)			−0.21 (−1.03, 0.61)			−0.29 (−1.13, 0.54)			−0.3 (−3.38, 2.77)			−2.49 (−28.46, 23.47)			−16.64 (−69.38, 36.1)		
Body mass index	0.02 (−0.05, 0.08)	0.60	0.01	0.04 (−0.03, 0.12)	0.24	0.03	0 (−0.02, 0.03)	0.90	0.00	0 (−0.02, 0.03)	0.84	0.001	−0.03 (−0.13, 0.08)	0.62	0.01	0.32 (−0.45, 1.09)	0.41	0.01	−0.23 (−1.72, 1.26)	0.75	0.002
Job type (Latko scale)		0.70	0.005		0.86	0.001		0.47	0.02		0.69	0.01		0.30	0.04		0.77	0.003		0.32	0.03
Non-repetitive (1–7 points on Latko scale)	Ref			Ref			Ref			Ref			Ref			Ref			Ref		
Repetitive (8–10 points on Latko scale)	0.14 (−0.59, 0.87)			−0.08 (−1, 0.85)			0.08 (−0.15, 0.31)			−0.05 (−0.28, 0.19)			−0.54 (−1.57, 0.5)			1.79 (−10.62, 14.19)			−10.4 (−31.21, 10.42)		
WHO-Five well-being index at baseline	−0.09 (−0.28, 0.11)	0.38	0.02	−0.06 (−0.29, 0.17)	0.59	0.01	−0.07 (−0.15, 0.01)	0.10	0.06	−0.08 (−0.16, 0.01)	0.09	0.10	0.05 (−0.29, 0.39)	0.77	0.002	/	/	/	0.3 (−5.79, 6.39)	0.92	0.00
Dominant hand affected		0.45	0.01		0.21	0.03		0.44	0.01		0.14	0.05		0.57	0.01		0.18	0.04		0.66	0.004
No	0.41 (−0.67, 1.49)			−0.76 (−1.97, 0.45)			−0.17 (−0.59, 0.26)			−0.34 (−0.8, 0.11)			0.47 (−1.21, 2.15)			−9.77 (−24.37, 4.82)			−6.62 (−36.49, 23.26)		
Yes	Ref			Ref			Ref														
Severity of CTS	/	/	/	/	/	/		0.69	0.01		0.52	0.03		0.16	0.07		0.21	0.06		0.52	0.03
Normal/mild	/	/	/	/	/	/	Ref			Ref			Ref			Ref			Ref		
Moderate	/	/	/	/	/	/	−0.09 (−0.53, 0.34)			−0.16 (−0.61, 0.28)			−0.28 (−1.95, 1.39)			−3.05 (−16.29, 10.19)			−8.22 (−36.92, 20.47)		
Severe	/	/	/	/	/	/	0.05 (−0.39, 0.48)			0.03 (−0.4, 0.46)			−1.27 (−2.96, 0.42)			6.28 (−7.49, 20.04)			−15.14 (−43.28, 13.01)		
Family history of CTS		0.39	0.01		0.04	0.08		0.36	0.02		0.19	0.04		0.01	0.13		0.003	0.16		0.87	0.001
No	Ref			Ref			Ref			Ref			Ref			Ref			Ref		
Yes	−0.35 (−1.17, 0.46)			−0.91 (−1.77, −0.05)			−0.14 (−0.45, 0.17)			−0.22 (−0.55, 0.11)			1.49 (0.34, 2.63)			−14.2 (−23.4, −5)			1.71 (−18.8, 22.23)		
Thumb opposition		0.53	0.01		0.27	0.02		0.41	0.01		0.14	0.10		0.19	0.04		0.99	0.00		0.72	0.003
No	Ref			Ref			Ref			Ref			Ref			Ref			Ref		
Yes	−0.25 (−1.06, 0.55)			−0.5 (−1.41, 0.41)			−0.12 (−0.41, 0.17)			−0.22 (−0.5, 0.07)			0.84 (−0.42, 2.11)			−0.04 (−10.49, 10.4)			3.76 (−17.16, 24.67)		
Presence of muscular atrophy in thenar area		0.53	0.01		0.84	0.001		0.03	0.10		0.07	0.10		0.88	0.001		0.01	0.13		0.01	0.13
No	Ref			Ref			Ref			Ref			Ref			Ref			Ref		
Yes	0.23 (−0.49, 0.95)			0.1 (−0.85, 1.04)			0.35 (0.04, 0.65)			0.3 (−0.03, 0.63)			−0.1 (−1.35, 1.15)			−12.98 (−23.1, −2.85)			−26.36 (−47.31, −5.42)		

Classification of partial eta-squared: 0.01 = small, 0.06 = medium, and 0.14 = large [24]

*NRS-PS* Perceived strength in the affected hand, *Ref* Reference category

Interestingly, the presence of muscular atrophy in the thenar area at baseline displayed medium size effects for multiple outcomes of CTS surgery: Patients suffering from muscular atrophy in the thenar area scored 0.35 (95 % CI [0.04, 0.65]) points higher in the BQ score of symptoms than patients without muscular atrophy, −12.98 [−23.1, −2.85] points lower in the WHO-Five well-being index, and −26.36 [−47.31, −5.42] points lower in the score of subjective quality of life. The effect size in the BQ score of function was also medium, but the regression coefficient was not statistically significant.

Similarly, there were medium to large effects of family history on DSL (−0.91 [−1.77, −0.05]), NRS-PS (1.49 [0.34, 2.63]), and the WHO-Five well-being index (−14.2 [−23.4, −5]). The WHO-Five well-being index itself as a measure of depressive symptoms at baseline played a rather marginal role with respect to surgery outcomes, with the exception of the functional BQ score. Age and sex had medium effects on NRS-PS.

The analysis of the predictors for sick leave was only possible for 42 cases providing this information. None of the univariable associations were significant, but there was some indication of variables which should become significant in a larger sample: type of work, severity of CTS, and whether the surgery affected the dominant hand (Table 5).

**Table 5:** Hazard ratio of return to work in univariable Cox regression (Hazard ratio > 1 means faster return to work)

	Hazard ratio (95 % confidence interval)	*p* value
Age (per 10 years)	1.24 (0.94, 1.62)	0.13
Sex		
Female	0.99 (0.51, 1.89)	0.97
Male	Ref	
Smoking status		
Non-smoker	Ref	
Current smoker	1.03 (0.52, 2.04)	0.93
Former smoker	1.25 (0.52, 3.01)	0.62
Qualification		
Student/in an apprenticeship	0.49 (0.12, 2.12)	0.34
Worker	0.74 (0.38, 1.42)	0.37
Employee	Ref	
Body mass index (per 1 kg/m^2^)	1.04 (0.99, 1.08)	0.11
Job type (Latko scale)		
Non-repetitive (1–7 points on Latko scale)	Ref	
Repetitive (8–10 points on Latko scale)	0.56 (0.29, 1.08)	0.09
WHO-Five well-being index (per 10 points)	1.05 (0.85, 1.31)	0.63
Dominant hand affected		
Yes	Ref	
No	1.62 (0.63, 4.18)	0.32
Severity of CTS		
Normal/mild	Ref	
Moderate	1.44 (0.65, 3.19)	0.37
Severe	1.71 (0.76, 3.87)	0.19
Family history of CTS		
No relative is affected	Ref	
One or more relatives are affected	1.15 (0.61, 2.17)	0.68
Thumb opposition		
Yes	0.76 (0.4, 1.46)	0.41
No	Ref	
Presence of muscular atrophy in thenar area		
Yes	1.77 (0.8, 3.9)	0.16
No	Ref	

*Ref* Reference category

## Discussion

In this prospective cohort, we studied the three and six month outcomes of CTS surgery. Overall, there was a positive effect of CTS surgery, with clinical outcomes already improving in the short term, while individual subjective outcomes improved in the long term. In terms of NCV, the improvement was proportional to the severity score at baseline. Most of the considered potential predictors did not display powerful effects on the outcomes, which conversely means that outcomes were similar for all cases and did not depend on individual characteristics. Depressive symptoms at baseline did not affect clinical or perceived outcomes negatively. Regarding the length of sick leave, there was some indication of associations, but these were not significant considering the limited sample size.

Positive effects of surgical CTS release were demonstrated in previous clinical studies, also in comparison to conservative therapy [2, 25]; our study is the first to confirm these findings for the outpatient setting in Germany. The time scale of improvement is different for immediate clinical as well as more subjective general outcomes, which is plausible. While the positive effects likely persist beyond the investigated initial six months, the true long-term effects could not be addressed in this study. However, studies with longer follow-up periods indicated a persistence of positive effects and no further improvement beyond six months [2, 26]. A recent review of a long-term follow-up after CTS surgery showed generally positive results for carpal tunnel release, with a clinical success rate between 75 and 90 % [27], suggesting a lasting positive impact of surgery.

The studied outcomes did not strongly depend on the investigated clinical and socio-demographic characteristics; equivalent to the observation that all patients benefited similarly from surgical treatment, not just those with specific characteristics. Clearly, given the limited sample size, our study was not designed to identify small effects; conversely small effects may not be clinically meaningful.

The severity score of CTS at baseline had a moderate negative impact on perceived strength (NRS-PS) and duration of sick leave – this could suggest that early diagnosis of CTS would be preferable in order to improve these outcomes. On the other hand, the severity of CTS based on NCV only had a small influence on more global subjective outcomes (well-being and quality of life), indicating some mismatch between NCV and the subjective improvement after surgery. Measurement of perceived strength on a continuous rating scale has not yet been standardized. Currently available is a wide range of instruments quantifying motor function, with several outcome measures considered necessary to capture the impact of a disorder like CTS on the individual. However, the use of multiple outcome measures places a substantial burden on the patient and the clinician [28], even if the additional costs of various instruments are not included. After all, an ideal and widely accepted measuring tool of motor function has still not been created. We therefore developed a subjective measure – a continuous numeric rating scale of perceived strength similar to a rating scale of pain –, a widely accepted method in quantifying individual levels of pain [29].

NCV at six months was proportional to the corresponding baseline value – this is consistent with the fact that more severe baseline values leave more residual impairment rather than allowing restitutio ad integrum (at least within the six months of follow-up). Other studies reported similar results of impaired nerve conducting velocity after long-term follow-up suggesting a compulsive inclusion of both functional and clinical results to assess the outcome after carpal tunnel release [30]. Despite being the only commonly accepted objective measure for CTS, the clinical importance of persisting abnormal distal latencies after surgery is a question yet to be answered [27].

Severity at baseline differed by sex, but otherwise sex was only associated with one outcome (NRS-PS) of surgery. These results are in line with other studies in which women report greater preoperative symptoms than men despite comparable or milder CTS grades on average underlying nerve conduction measurement results [10, 13]. As in many other syndromes and diseases, for a given clinical severity, women reported more severe symptoms. Current findings regarding sex differences in experimental pain indicate greater pain sensitivity among females compared to males for most pain modalities, likely influenced by a variety of social and psychological processes, gonadal hormones, endogenous pain modulatory systems, gender roles, and cognitive/affective factors (for review see [29]). However, one must consider that women are overrepresented in jobs with high risks for CTS [3, 31]. This could lead to an increased subjective stress caused by the repeating provocation of symptoms during job demands and daily activity.

### Strengths and limitations

The strengths of the study lie in its prospective design with a standardized clinical and electrophysiological examination. The study reports experience from a single outpatient clinic performing CTS surgery. While we are not aware of specific differences, the results might not be generalizable to other settings. There was also a substantial drop out rate among participants between the baseline and the follow-up at three or six months. While this does not affect the observed effects on the individual level, the extrapolation of the findings to all CTS patients might be restricted.

## Conclusions

There is a significant improvement of clinical and subjective outcomes after CTS surgery in the outpatient sector – the improvement is largely independent of socio-demographic and clinical characteristics of the patients.

### Ethics approval and consent to participate

The study was approved by the Regional Ethical Review Board, University of Münster. All patients provided written informed consent before entering the study.

### Availability of data and materials

The dataset supporting the conclusions of this article is available from the corresponding author.

## Abbreviations

ANCOVA: analysis of covariance
BMI: body mass index
BQ: Boston questionnaire
CI: confidence interval
CTS: carpal tunnel syndrome
DML: distal motor latencies
DSL: distal sensory latencies
NCV: nerve conduction velocity
NRS-P: numeric rating scale of pain
NRS-PS: numeric rating scale of perceived strength
